# Seizure Forecasting from Idea to Reality. Outcomes of the My Seizure Gauge Epilepsy Innovation Institute Workshop

**DOI:** 10.1523/ENEURO.0349-17.2017

**Published:** 2017-12-27

**Authors:** Sonya B. Dumanis, Jaqueline A. French, Christophe Bernard, Gregory A. Worrell, Brandy E. Fureman

**Affiliations:** 1 Research and New Therapies, Epilepsy Foundation of America, Landover, MD 20784; 2 Department of Neurology, New York University, New York, NY 10003; 3 Aix Marseille University, INSERM, INS, Inst Neurosci Syst, Marseille, 13005, France; 4 Mayo Systems Electrophysiology Laboratory, Mayo Clinic, Rochester, MN 55902

**Keywords:** algorithm, data collection, epilepsy, multimodal input, seizure forecasting, temporal pattern

## Abstract

The Epilepsy Innovation Institute (Ei^2^) is a new research program of the Epilepsy Foundation designed to be an innovation incubator for epilepsy. Ei^2^ research areas are selected based on community surveys that ask people impacted by epilepsy what they would like researchers to focus on. In their 2016 survey, unpredictability was selected as a top issue regardless of seizure frequency or severity. In response to this need, Ei^2^ launched the My Seizure Gauge challenge, with the end goal of creating a personalized seizure advisory system device. Prior to moving forward, Ei^2^ convened a diverse group of stakeholders from people impacted by epilepsy and clinicians, to device developers and data scientists, to basic science researchers and regulators, for a state of the science assessment on seizure forecasting. From the discussions, it was clear that we are at an exciting crossroads. With the advances in bioengineering, we can utilize digital markers, wearables, and biosensors as parameters for a seizure-forecasting algorithm. There are also over a thousand individuals who have been implanted with ambulatory intracranial EEG recording devices. Pairing up peripheral measurements to brain states could identify new relationships and insights. Another key component is the heterogeneity of the relationships indicating that pooling findings across groups is suboptimal, and that data collection will need to be done on longer time scales to allow for individualization of potential seizure-forecasting algorithms.

## Significance Statement

Unpredictability of seizures is a top issue for those living with epilepsy regardless of seizure frequency and type. There is the fear of not knowing when a seizure will start and not knowing what triggers the seizure onset. In August, the Epilepsy Innovation Institute (Ei^2^) convened a diverse group of stakeholders to assess the state of the science on seizure-forecasting algorithms. Seizure forecasting shifts away from categorical seizure prediction assessments of whether a seizure will or will not occur and instead focuses on identifying the brain state wherein there is a high probability of a seizure occurrence. Here, we discuss the outcomes of those discussions and next steps.

## Introduction

Epilepsy is a common neurologic condition characterized by the occurrence of recurrent spontaneous seizures. The World Health Organization estimates that there are over 50 million people living with epilepsy worldwide (World Health Organization, 2017). About a third of people living with epilepsy do not have seizure control, and those whose seizures are controlled are at risk of breakthrough seizures (Brodie et al., 2012). This staggering number has not changed in decades, despite over 14 new therapies for epilepsy entering the market since the 1990s (Löscher and Schmidt, 2011)

In 2016, the Epilepsy Innovation Institute (Ei^2^), a research program of the Epilepsy Foundation, released an online survey asking their community what aspects of epilepsy impact them the most. Over one thousand individuals responded from across the United States and abroad. An overwhelming majority of respondents, regardless of seizure frequency and type, selected unpredictability of seizures as a top issue (Epilepsy Foundation, 2016). Many wrote about the fear of not knowing when a seizure will start and not knowing what triggers the seizure onset. In response to this survey, Ei^2^ developed the My Seizure Gauge initiative with the end-goal of creating a seizure risk-assessment system that could evaluate the likelihood of a seizure on a daily basis. Before moving forward with the initiative, Ei^2^ hosted an innovation workshop to assess the state of the science on seizure-forecasting and risk assessment algorithms. Here, we are defining seizure forecasting as the process of identifying body states wherein there is a high probability of a seizure occurrence.

The following scientific themes emerged from the discussions. (1) Seizures have multi-temporal patterns on ultradian, circadian, and multi-day time scales. (2) Multimodal analysis of seizure events coupling EEG with non-EEG measures may enhance seizure-forecasting algorithms. (3) Individualization and personalization of a seizure-forecasting algorithm is necessary. Each of these themes is highlighted in more detail below.

## Seizures Have Multi-Temporal Patterns on Ultradian, Circadian, and Multi-Day Time Scales

An overwhelming body of evidence indicates that seizures have nonrandom time-specific patterns (Langdon-Down and Russell Brain, 1929; Griffiths and Fox, 1938; Bercel, 2006; Loddenkemper et al., 2011). Recently, these observations have been replicated in long-term ambulatory intracranial recordings from people implanted with the NeuroVista device (Cook et al., 2013; Karoly et al., 2016, 2017), and in the Neuropace Rapid Neurostimulation (RNS) device (Spencer et al., 2016; Baud et al., in press). Ninety-eight percent of people with an implanted Neuropace RNS device have clear circadian and/or ultradian patterns for electrocordiographic seizures (Spencer et al., 2016). In addition to circadian rhythms, researchers also observed multi-day cycle of interictal epileptiform activity varying between 7 and 35 d across patients, but relatively stable within each patient (Baud et al., in press).

Interestingly, in both the 1938 Griffiths and Fox study as well as in the recent Neurovista and Neuropace studies, the complexity of detecting time patterns is discussed (Griffiths and Fox, 1938; Freestone et al., 2017; Baud et al., in press). Across the whole group, there was a lot of variability, but within an individual, seizure time patterns could be very consistent. Understanding these brain rhythms, why they happen and how they can influence seizure occurrences may be key to understanding seizure susceptibility for the individual, and thus to developing a personalized therapeutic strategy.

## Multimodal Analysis of Seizure Events Coupling EEG with Non-EEG Measures May Enhance Seizure-Forecasting Algorithms

The brain is a dynamic organ reacting to internal and external inputs. The temporal rhythm of seizures suggests that there may be several metabolic or biophysical measures that could be detected before a seizure event. For example, several biophysical parameters are suggested to change slowly during or preceding a seizure including extracellular levels of potassium, oxygen, pH, and intracellular NADH/FAD+ (Jirsa et al., 2014). Very fast oscillations (VFOs) have also been observed to precede seizure onset, and a review of the literature suggests that their occurrence may be due to gap junctions that are brain pH dependent (Traub et al., 2010).

With advances in bioengineering, we have the capabilities to measure ionic changes *in vivo* coupled with intracranial EEG recordings. A recent study demonstrated that changes in the extracellular composition of potassium, calcium and magnesium independent of local electrical activity could distinguish which rodents were in a sleep brain state versus an awake brain state (Ding et al., 2016). This study highlights how measuring brain ionic changes *in vivo* could enhance our understanding of seizure vulnerable brain states.

There may also be multiple ways to capture information about an individual noninvasively that were previously impossible. In 2017, Mike Snyder’s group provided the proof of principle for how commercially wearable biosensors could identify early signs of Lyme disease and inflammatory responses (Li et al., 2017). With video and 3D imaging analysis, we are also now capable of mapping subsecond units of movement that are indiscernible to the human eye to analyze behavior (Wiltschko et al., 2015). Indeed, sweat sensing technologies have advanced rapidly in the past five years. It will soon be possible to have noninvasive continuous monitoring of various metabolites such as cortisol, something that was not possible previously (Bandodkar and Wang, 2014; Rose et al., 2015; Sonner et al., 2015). There are also optical measures of motion and stress that can recognize heart rate and respiration at a distance (Nam et al., 2016). These tools could be used to analyze potential physiologic and behavioral changes occurring hours before a seizure event.

Sheryl Haut’s group has reported that a subset of people living with epilepsy are very good at predicting their seizures up to 6 hours before the seizure event occurs (Haut et al., 2013). These individuals kept a diary and reported premonitory features associated with accurate predicted seizure occurrence. The top ten features included blurred vision, light sensitivity, dizziness, feeling emotional, concentration difficulty, hunger/food cravings, noise sensitivity, tiredness/weariness, thirst, and difficulty with thoughts. This all suggests that there may be alterations in body chemistry, associated behaviors, and symptoms that could improve seizure forecasting. Some of these body changes could be picked up through existing biosensors, mobile devices, or video monitoring. There are also preliminary findings reported by Dean Freestone, University of Melbourne, that atmospheric change such as humidity and pressure may be a variable in seizure likelihood for people with epilepsy. This intriguing observation suggests that the surround environments may also play a role in the analysis. At the Ei^2^ workshop, multiple parameters were identified as potential measurements to consider in addition to EEG recordings when thinking about creating a seizure prediction device (Table 1).

**Table 1.** Potential measurements discussed at the Ei^^2^^ workshop that could enhance a seizure-forecasting algorithm

• Mood*	• Stress*	• Compliance
• Cortisol	• Fatigue*	• Illness*
• Orexin	• Irritability*	• Food/alcohol intake
• Patient self-prediction*	• Sex hormones	• Orientation (cognitive)
• Electrical dermal activity	• pH (brain)	• Gait
• Heart rate	• Time of day*	• Finer movements
• Temperature/weather	• Antiepileptic drug levels	• Ketones
• Respiration	• Blood oxygen	• Speech
• Sleep cycle changes (sleep/wake staging)	• Inflammatory markers	• Body Temperature
• Sleep quality	• Glucose	
	• External environment	

These measurements could be collected in numerous ways. An asterisk indicates those that could be captured by patient diary, others could be measured through smartphone, biosensors, or through sweat collection.

With the advances in bioengineering and biosensors, we have the capability to acquire noninvasive multimodal data that allow us to identify potential lead candidate signals that inform about seizure probability to circle back and test.

## Individualization and Personalization of a Seizure-Forecasting Algorithm Is Necessary

There is a complexity and heterogeneity to understanding the susceptibility of seizures. The International League Against Epilepsy (ILAE) has stratified the underlying causes for epilepsy into six categories: genetics, brain structure abnormalities, metabolism changes, immune system abnormalities, infectious disease, and unknown causes (Berg and Millichap, 2013). Not only are there multiple causes for a seizure, there are also varying responses to those causes within an individual that could lead to a seizure vulnerable brain state. Therefore, pooling data across individuals becomes suboptimal. The need for individualization also underscores the need for longitudinal data. Seizures are episodic events, and there needs to be enough seizures for the algorithms to be optimized over time.

Previously, we only had short-term intracranial EEG data (typically up to one week) for analysis from presurgical monitoring units (Mormann et al., 2007). The short-term recordings are of too limited a time span with insufficient interictal and ictal data to build patient-specific models for seizure likelihood. There are now over a thousand individuals who have ambulatory intracranial EEG systems through the FDA approved Neuropace RNS system or through the Activa PC system by Medtronic in clinical trials. There are also less invasive seizure monitoring devices in development from ambulatory surface EEG caps to subscalp EEG implants. This allows us to have access to real-time longitudinal data (on the magnitude of years) of EEG recordings. We can use these devices to link measured brain states with peripheral measures to improve our ability to assess seizure likelihood.

One approach could be to use machine learning. Deep learning has proven to be highly successful at automated complicated pattern recognition tasks in EEG (Nurse et al., 2016; Kiral-Kornek et al., 2017) and multimodal data and, therefore, constitutes a generalizable technique for a seizure prediction system that can be tuned to an individual’s unique seizure data signature. Machine learning is not the answer for all problems, but it works well with unstructured data. However, for such an approach to be meaningful, subject matter expertise will be critical to ensure accurate classifications of the data and interpretable results. For example, mathematical modeling of electrophysiological signatures of seizures evolutionarily conserved across species from flies to humans has yielded 16 distinct electrocardiographic seizure profiles (Jirsa et al., 2014). This new taxonomy may spur potential new insights into seizure mechanism that could help interpret the data findings and find correlations in body chemistry associated with these different seizure classifications. Moreover, insights into seizure onset mechanisms from a dynamical systems perspective may help identify useful data features (Meisel and Kuehn, 2012) to integrate into machine learning algorithms.

Once an algorithm is developed, it will also be important to consider the ability to personalize the algorithm. There are different utilities for knowing when someone is at risk for seizures. For example, some individuals may want to know when they are likely to have a subclinical seizure (an electrographical seizure without any outward symptoms) while others may only want to know when they are likely to be experiencing a tonic-clonic seizures (convulsions) or loss of consciousness. Some are troubled more than others by false positive warnings, which can elevate anxiety levels. There should also be considerations about what forecasting ranges are useful and what forecasting probabilities would be meaningful. Lessons learned from the Neurovista trial were that, although it was a mathematically sound way to characterize performance, a patient might have a different assessment of what a good performance algorithm means. Therefore, for any algorithm, a patient feedback loop is critical to ensure specificity of the algorithm, successful adoption and good performance. One of the workshop participants likened it to a Pandora Music algorithm, where the user would hit like or don’t like to the forecasting to ensure that the forecasting algorithm could be optimized and fine-tuned to the individual.

## Next Steps

The overarching goal of Ei^2^ is to lead an effort that would create an individualized seizure gauge that will allow a person with epilepsy to monitor the likelihood of a seizure on a daily basis. The word likelihood is emphasized as it shifts the focus from 100% certainty to assessing probability states. Anecdotally, there are some patients who report that after decades of living with epilepsy they can know when they are likely to have a seizure event. Our goal is to speed that process up for the community. From the Innovation Workshop, it became clear that we need to focus on identifying and better understanding the changes in the body that may precede the onset of a seizure, at a time course that may be hours or days before the clinical (observable) seizure. Therefore, linking an ambulatory long-term seizure monitoring approach (from already implanted in-depth intracranial EEGs, subscalp EEGs, to wearable surface EEG caps, or video monitoring) to a host of non-EEG-based methods from emerging biosensors, wearable device technology, and digital markers on a longitudinal time scale would help us identify new relationships between brain state and noninvasive or minimally invasive readouts. Insights from this study can then be used to design less invasive approaches to a future seizure gauge device for forecasting seizures.
